# Quality of recovery after day care surgery with app-controlled remote monitoring: study protocol for a randomized controlled trial

**DOI:** 10.1186/s13063-023-07121-6

**Published:** 2023-02-09

**Authors:** B. Thiel, M. B. Godfried, M. E. van Emst, L. M. Vernooij, L. M. van Vliet, E. Rumke, R. T. M. van Dongen, W. Gerrits, J. S. H. A. Koopman, C. J. Kalkman

**Affiliations:** 1 Department of Anaesthesiology, OLVG Hospital (Oost), Amsterdam, 1090 HM the Netherlands; 2 Department of Anaesthesia and Intensive Care, University Medical Centre Utrecht (UMCU), Utrecht, 3508 GA The Netherlands; 3 University Leiden, Wassenaarseweg 52, Leiden, 233 AK the Netherlands; 4 Department of Anaesthesiology, Canisius Wilhelmina Hospital (CWZ), Weg door Jonkerbos 100, Nijmegen, 6532 SZ The Netherlands; 5 Department of Anaesthesiology, Maasstad Ziekenhuis, Maasstadweg 21, Rotterdam, 3079 DZ The Netherlands

**Keywords:** Day care surgery, Postoperative pain, Postoperative nausea, Postoperative recovery, Remote monitoring, eHealth, Smartphone application, Randomized controlled trial

## Abstract

**Background:**

The majority of surgical interventions are performed in day care and patients are discharged after the first critical postoperative period. At home, patients have limited options to contact healthcare providers in the hospital in case of severe pain and nausea. A smartphone application for patients to self-record pain and nausea when at home after day care surgery might improve patient’s recovery. Currently patient experiences with smartphone applications are promising; however, we do not know whether remote monitoring with such an application also improves the patient’s recovery. This study aims to evaluate the experienced quality of recovery after day care surgery between patients provided with the smartphone application for remote monitoring and patients receiving standard care without remote monitoring.

**Methods:**

This non-blinded randomized controlled trial with mixed methods design will include 310 adult patients scheduled for day care surgery.

The intervention group receives the smartphone application with text message function for remote monitoring that enables patients to record pain and nausea. An anaesthesia professional trained in empathetic communication, who will contact the patient in case of severe pain or nausea, performs daily monitoring. The control group receives standard care, with post-discharge verbal and paper instructions.

The main study endpoint is the difference in perceived quality of recovery, measured with the QoR-15 questionnaire on the 7th day after day care surgery. Secondary endpoints are the overall score on the Quality of Recovery-15 at day 1, 4 and 7-post discharge, the perceived quality of hospital aftercare and experienced psychological effects of remote monitoring during postoperative recovery from day care surgery.

**Discussion:**

This study will investigate if facilitating patients and healthcare professionals with a tool for accessible and empathetic communication might lead to an improved quality of the postoperative recovery period.

**Trial registration:**

The ‘Quality of recovery after day care surgery with app-controlled remote monitoring: a randomized controlled trial’ is approved and registered on 23 February 2022 by Research Ethics Committees United with registration number R21.076/NL78144.100.21. The protocol NL78144.100.21, ‘Quality of recovery after day care surgery with app-controlled remote monitoring: a randomized controlled trial’, is registered at the ClinicalTrials.gov public website (registration date 16 February 2022; NCT05244772)

**Supplementary Information:**

The online version contains supplementary material available at 10.1186/s13063-023-07121-6.

## Administrative information

**Table:** 

Title {1}	Quality of Recovery after day care surgery with app controlled Remote Monitoring: a randomized controlled trial protocol. QuReMo trial
Trial registration {2a and 2b}	Medical Research Ethics Committees United (MEC-U) registration number R21.076 / NL78144.100.2, Postal Code 2500, 3440 EM Nieuwegein, info@mec-u.nl, www.mec-u.nl Clinical trials.gov identifier: NCT05244772
Protocol version {3}	Protocol version 5, 22-06-2022
Funding {4}	SIDN fund, a public benefit organization for Dutch internet domain registration, partially funded writing and submitting the study protocol. Furthermore, they will contribute to the analysis and writing the final manuscript. The funders had no content-related role in the study design, data collection, analysis, decision to publish or preparation of the manuscript.
Author details {5a}	B.Thiel Position: Physician assistant / Clinical epidemiologist Role: trial coordinator / PhD student Department of anesthesiology OLVG Hospital (Oost) Mailbox 95500 Postal Code 1090 HM Amsterdam, the Netherlands e-mail: b.thiel@olvg.nl M.B. Godfried Position: Anaesthetist Role: Principal investigator OLVG Department of anaesthesiology OLVG Hospital (Oost) Mailbox 95500 Postal Code 1090 HM Amsterdam, the Netherlands e-mail: m.b.godfried@olvg.nl M.E. van Emst Position: resident anaesthesiology Role: Investigator Department of anaesthesiology OLVG Hospital (Oost) Mailbox 95500 Postal Code 1090 HM Amsterdam, the Netherlands e-mail: m.e.vanemst@olvg.nl L.M. Vernooij Position: Clinical Epidemiologist Role: Investigator Department of anaesthesia and intensive care University Medical Centre Utrecht (UMCU) Mailbox 85500 Postal Code 3508 GA Utrecht, the Netherlands e-mail: L.M.Vernooij@umcutrecht.nl L.M. van Vliet Position: Psychologist/ Researcher Role: Investigator University Leiden Wassenaarseweg 52 Postal Code 233 AK Leiden, the Netherlands e-mail: l.m.van.vliet@fsw.leiden univ.nl E. Rumke Position: trainee psychologist Role: Investigator University Leiden Wassenaarseweg 52 Postal Code 233 AK Leiden, the Netherlands W. Gerrits Position: Anaesthetist / Pain specialist Role: Coordinating Investigator CWZ Department of Anaesthesiology Canisius Wilhelmina Hospital (CWZ) Weg door Jonkerbos 100 Postal Code 6532 SZ Nijmegen, the Netherlands e-mail: w.gerrits@cwz.nl R.T.M. van Dongen Position: Anaesthetist / Pain specialist Role: Principal Investigator CWZ Department of Anaesthesiology Canisius Wilhelmina Hospital (CWZ) Weg door Jonkerbos 100 Postal Code 6532 SZ Nijmegen, the Netherlands e-mail: r.t.m.vandongen@cwz.nl J.S.H.A. Koopman Position: Anaesthetist Role: Principal Investigator Maasstad Hospital Department of Anaesthesiology Maasstad Ziekenhuis Maasstadweg 21 Postal Code 3079 DZ Rotterdam the Netherlands e-mail: KoopmanJ@maasstadziekenhuis.nl C.J. Kalkman Position: Professor anaesthesiology Role: Project Supervisor Department of anaesthesia and intensive care University Medical Centre Utrecht (UMCU Mailbox 85500 Postal Code 3508 GA Utrecht, the Netherlands e-mail: c.j.kalkman@umcutrecht.nl
Name and contact information for the trial sponsor {5b}	B. Thiel Department of anesthesiology OLVG Hospital (Oost) Mailbox 95500 Postal Code 1090 HM Amsterdam, the Netherlands e-mail: b.thiel@olvg.nl telephone: +31 20 5999111
Role of sponsor {5c}	The coordinating center of the trail OLVG is responsible for primary design; data collection, management, analysis and interpretation of data, writing the report and the decision to submit the report for publication.

## Introduction

### Background and rationale {6a}

Improving high quality and cost-effective healthcare for outpatients is a hospital priority. The volume and complexity of outpatient surgical procedures continues to increase, with a wider range of patients now considered suitable for day care surgery. In the Netherlands, 54% from all surgical procedures annually are performed in day care, and this is expected to increase [1, 2]. To date, the majority of day care surgical patients are being discharged a few hours after the critical post-operative phase is passed, and vital signs and physical wellbeing have been stabilized [3, 4]. Most hospitals provide their patients with verbal and paper instructions in case of severe pain nausea and alleged surgical complications. The usual advice is to contact the surgical or anaesthesia outpatient department during office hours. If unavailable, patients are advised to contact their general practitioner or the hospitals’ emergency department. For patients in pain, nauseated or worried about physical wellbeing, contacting the hospital can be a challenging task. In addition, doctors and nurses have ongoing clinical obligations, which makes it unrealistic to expect that they are available on-demand. As moderate-to-severe postoperative pain [5, 6] and nausea [7–10] is still common after surgery, an easy eHealth tool for the patient to communicate with a health care professional seems a crucial element of after care. EHealth interventions are currently broadly applied in perioperative care (e.g. remote monitoring, educational websites and tele-rehabilitation) [11, 12]. They have proven to be an easy and an approachable source of information for patients and a reliable aid for their follow-up [11]. Due to eHealth’s technical possibility of direct and empathetic contact, patient’s recovery could improve. Previous studies researching patient and healthcare professional communication show health benefit for patients receiving empathetic care [13, 14]. Moreover, results from previous studies show a significant reduction of hospital admissions, days spent in hospital and healthcare costs [15, 16]. Despite the promising results with a similar application amongst clinically admitted patients, its effect on patient’s recovery after day care surgery is unknown [17].

Therefore, we configured an existing and already applied remote monitoring smartphone application to enable follow-up of patients after day care surgery. This application records postoperative pain and nausea and can be used to contact a healthcare professional. Anaesthesia healthcare professionals specially trained in empathetic contact report back to the patient either by text message of telephone.

We hypothesized that the realization of being monitored remotely and receiving empathetic feedback from healthcare professionals during the postoperative recovery period will improve patient experienced recovery. To study this, we will perform a non-blinded randomized controlled trial with mixed methods

### Objectives {7}

Primary objective

To assess the difference in perceived quality of recovery after day care surgery between patients with or without a smartphone application for remote monitoring, measured with the QoR-15 questionnaire on the seventh day after day care surgery

Secondary objective(s)

To assess the experienced postoperative pain (POP) between patients provided with a smartphone application for remote monitoring and patients receiving standard of care (no remote monitoring) after day care surgery

To assess the experienced post discharge nausea and vomiting (PDNV) between patients provided with a smartphone application for remote monitoring and patients receiving standard of care (no remote monitoring) after day care surgery

To assess the difference in the number of surgical complications rated with the Clavien-Dindo classification of surgical complication between patients provided with a smartphone application for remote monitoring and patients receiving standard of care (no remote monitoring) after day care surgery

To assess the difference in the number of re-admissions between patients provided with a smartphone application for remote monitoring and patients receiving standard of care (no remote monitoring) after day care surgery

To assess the difference in the number of hospital contacts/general practitioner contacts between patients provided with a smartphone application for remote monitoring and patients receiving standard of care (no remote monitoring) after day care surgery

To assess the difference in experienced quality of communication with the hospital staff between patients provided with a smartphone application for remote monitoring and patients receiving standard of care (no remote monitoring) after day care surgery

To assess the difference in experienced psychological effects between patients provided with a smartphone application for remote monitoring and patients receiving standard of care (no remote monitoring) after day care surgery

To assess experienced quality of communication with the hospital staff and experienced psychological effect of patients using the remote monitoring application and patients not using the remote monitoring application

### Trial design {8}

This protocol is a two-arm multicentre non-blinded randomized controlled trial with mixed methods design that will be conducted at the day care nursing wards of OLVG Hospital (Locations Oosterpark and Jan Tooropstraat), Amsterdam, the Netherlands, Canisius Wilhelmina Hospital, Nijmegen, the Netherlands and Maasstad Hospital, Rotterdam, the Netherlands. Surgical day care patients will be randomly assigned to one of the two study arms, remote monitoring with a smartphone application (intervention) or standard of care (no remote monitoring) after hospital discharge. Study period is expected to run from first of February 2022 up to 31 December 2022.

## Methods: participants, interventions and outcomes

### Study setting {9}

For this study, we will enrol 310 patients who will undergo a surgical intervention in day care at one of the participating general hospitals. This trial was designed in accordance with the Standard Protocol Items: Recommendation for Interventional Trails (SPIRIT) guidelines for clinical trials [18].

### Eligibility criteria {10}

In order to be eligible to participate in this study, a patient must meet all of the following criteria:

- Age: older than or equal to 18 years
- Pre-anaesthesia conclusion: ASA I to III [19]
- Scheduled for day care surgery for one of the following surgical specialties: gynaecology, eye, ear-nose-throat, oral and maxillofacial, orthopaedics, general and vascular, trauma and urology
- In possession of a smartphone with operating system iOS 14.0 or later or Android 7.0 or later

A potential patient who meets any of the following criteria will be excluded from participation in this study:

- Not able to speak or understand the Dutch language
- Mentally impaired (e.g. dementia, retardation)
- Not able or willing to sign informed consent

If a patient is unexpectedly admitted after surgery (and randomization), they are excluded and replaced.

### Who will take informed consent? {26a}

An anaesthesiologist, trainee nurse specialist, physician assistant or medical assistant will verbally explain the study during anaesthesia pre assessment (i.e. telephone assessment or during the visit at the anaesthesia outpatient clinic). If interested, they receive an information-letter about the study and an informed consent form (Supplemental material: PIF_IC_e1_e2_QuReMo_V4_22_6_2022).

Patients who are not informed during the preoperative assessment will be contacted afterwards by one of the researchers to explain the study. If interested, they receive an information-letter about the study and an informed consent form. After a reflection period of 48 h, patients are contacted by one of the researchers by telephone and asked if they will participate. All eligible patients are earmarked in the electronic patient record. If not willing or able to participate stated reasons are recorded. Informed consent forms are collected on the day of admission and both researchers and patients receive a wet ink copy of the consent form

### Additional consent provisions for collection and use of participant data and biological specimens {26b}

Not applicable

### Interventions

#### Explanation for the choice of comparators {6b}

Patient will have a pre-anaesthesia workup (e.g. telephone assessment or an assessment during a physical visit to the anaesthesia outpatient department) according to the standards of the Dutch association of Anaesthesiologist and Federation of Medical Specialists [20]. During the admission, patients receive perioperative anaesthesia care according to standards based on the Guidelines for treatment of the Dutch association of anaesthesiologists [20, 21].

Either nurses or doctors, at least once per perioperative phase (e.g. recovery and day care ward), regularly ask the patient about their postoperative pain and nausea. In case of severe pain, the patient is administered paracetamol, NSAIDs, tramadol or opioids. In case of nausea, the patient is administered antiemetic drugs according to the latest guidelines, starting with granisetron as first step [22]. Postoperative medication is ordered and supervised by the attending anaesthetist.

Before discharge, patients receive verbal and paper (Supplemental material: k6_discharge information_Dutch) care and recovery instructions from a day care ward nurse. In addition, they receive a medication box containing paracetamol, non-steroid inflammatory drug (NSAID), metoclopramide a proton pump inhibitor (if indicated) and opioids (if indicated) this according to the guideline of the Dutch National association of anaesthetists (NVA) and in line with the World Health Organization pain ‘ladder’ [21, 23]

#### Intervention description {11a}

In addition to standard care as delineated above, the intervention group (remote monitoring after discharge) receives 2 days before admission an invitation e-mail from Luscii © sent by an anaesthesia medical assistant with instructions to download and install the remote monitoring application. Once back home after discharge, patients can start using the smartphone application for recording pain and nausea and ask additional questions about their recovery. The patient can record at any time when they feel the necessity to do so; moreover, once a day, the patient receives a push notification to record pain and nausea. The application is operational up to 7 days post discharge. After this period, the application is disconnected from the Luscii © server.

Recording with the application starts with a daily notification at 10:00 am with the question if the patient is in pain or nauseous. The patient is requested to record pain and nausea present at that moment. If the patient is in pain, the following additional questions must be answered: Is your pain bearable? Are you hindered by pain? Do you want something done to relief your pain? Additionally, the patient can rate the pain on a faces scale from 0 to 10. If the patient is nauseous the following additional questions must be answered; are you hindered in daily activities being nauseous, did you experience any dry retching or vomiting?, are you using medication for relief?, do you want something done to relief your nausea, followed by the question for additional comments. Subsequently, pain and nausea recordings and additional comments will appear as an alert in the electronic back office systems of Thuismeten (Luscii) ©.

The alerts are set to the in-app questions ‘do you want something done to relief your pain or nausea?’ or an increase of 3 points numerical rating scale (NRS) for pain and additional questions asked in the apps messaging service. In case of recording an alert, an anaesthesia medical assistant will contact the patient during office hours from 08:00 to 16:30 by using the applications’ messaging service or by telephone. Participants are explained by the researchers during recruitment and day care nurses at discharge that the messaging is not an on demand service. If they experience an emergency they are instructed to contact the hospital emergency department or the National emergency number, depending on the urgency.

The medical assistant will follow a protocolled workflow to assess the required actions (Supplemental material: Decision tree remote monitoring). If contacting the patient by telephone is necessary, the medical assistant will use a call script to assess patients’ pain, nausea and physical status (call script remote monitoring). The medical assistant will provide the patient appropriate and empathetic feedback by the message service or during a telephone call. In case of a possible medical alert or a necessary medication adjustment, the medical assistant will consult the anaesthetist, trainee nurse specialist or physician assistant responsible for remote postoperative pain care.

The remote monitoring application is a configuration of an already existing and applied monitoring platform by Luscii Healthtech B.V. The Luscii tele-monitoring platform is currently being used in the care for patients with acute and chronic diseases such as hypertension during pregnancy, hearth failure, COPD and COVID-19. The different telemonitoring modules are based on a direct feedback loop between patients and health care providers and alarming combinations of patient recorded results [24, 25]. The configuration for postoperative monitoring was commissioned by OLVG hospital. During this study versions 2.43.0 or higher are used. The application is CE IIa certified. For more information about Luscii technical, privacy and security specifications [26, 27].

The involved healthcare professionals who will respond to patients’ app-completion will be trained in empathic behaviours by an academic expert on the topic (LV, supported by ER). The specific behaviours they are being taught include the following: (i) providing reassurance for continued care (e.g. ‘you can always contact us via the app’ [28], (ii) responding to emotions using the NURSE framework (Name emotion, show Understanding, show Respect, show Support, Explore emotions; e.g. ‘I understand this is really tough for you’) [29–31]; (iii) showing interest in the patient as person behind their illness (e.g. ‘are you being taken well-care off’? [32]. Based on clinical experience, providers were also instructed to start with an open question and let patients talk for a bit; wish patients well; to also respond to positive app-scores, so patients know there is always someone available in case the situation changes; and to also honestly confess if you don’t have the answers to certain questions but to follow-up on these.

#### Criteria for discontinuing or modifying allocated interventions {11b}

Patients can leave the study without any consequences at any time for any reason if they wish to do so. The investigator can decide to withdraw a patient from the study for urgent medical reasons. These include patients who experience an unexpected post-operative complication or prolonged recovery with overnight hospital stay. Patients staying overnight unexpectedly will be excluded from the study and replaced.

#### Strategies to improve adherence to interventions {11c}

Participating in this study does not pose any additional risks for patients. Patients in the remote monitoring group are being asked to use a smartphone application to record pain and nausea daily for up to 7 days post discharge. Recording pain and nausea will take 2 min daily. Patients from both groups are being asked to fill in the validated QoR-15 questionnaire 1 day before admission and on the 1st, 4th and 7th day post discharge, and this will take 2.5 min per questionnaire. This questionnaire is sent to the participant by e-mail using the services of Castor EDC © and can be completed with an electronic link. No extra hospital visits, physical examinations or tests are required. In addition, patients who have given permission for an interview to assess their recovery period and the quality of communication with the hospital receive a telephone appointment for a 30-min semi-structured interview with researcher ER. Patients in the remote monitoring group could benefit from participating in the study because they are monitored daily by a healthcare professional. Therefore, severe pain, nausea and possibly other complications can be noticed and managed earlier.

#### Relevant concomitant care permitted or prohibited during the trial {11d}

There is no restriction to access or use of care during the trial for participating patients.

#### Provisions for post-trial care {30}

Regarding the design of this study, post-trial care is not considered necessary as no harm is to be expected from participating in this trial.

### Outcomes {12}

#### Main study outcome

The main study outcome is quality of recovery measured with the Quality of Recovery questionnaire-15 (QoR-15) [33, 34] on the 7th day after day care surgery (Supplemental material: f1_questionnaire_V1-16-7-2012).

#### Secondary study outcome

Postoperative pain: experienced postoperative pain (POP) assessed with (in-app) NRS for pain [35] (combined with 11 faces scale)

Post discharge nausea and vomiting (PDNV): experienced PDNV measured with the Myles’ PDNV assessment scale [21] between intervention and non-intervention group at day 1, 4 and 7 post discharge

Medication dosage and use: the total dosage of administered analgesics will be assessed during hospital stay and recorded in the medical record. The total dosage of administered analgesics at home after discharge will assessed by asking patients to indicate which pain medication they have been using at day 1, 4 and 7 post discharge

The number of contacts with the hospital, general practitioner or emergency department. All types of contacts (e.g. physically, e-mail, telephone) will be assessed by reviewing the PDMS for registered contacts and by asking the patients to indicate the number of contacts, measured at day 1, 4 and 7-post discharge

The number of (surgical) complications assessed according to the Clavien-Dindo classification of surgical complications [36, 37] will be assessed by reviewing the PDMS and by asking the patients to indicate the number of complications, measured at day 1, 4 and 7 post discharge

The number of readmissions. A readmission is defined as an admission to hospital or emergency department for medical care or observation regarding their surgical intervention. Readmissions will be assessed by reviewing the PDMS and by asking the patient to indicate the number of readmissions. Measured at day 1, 4 and 7 post discharge

Satisfaction: patients’ satisfaction with the provided care will be assesses using a 1-item self-created VAS scale (‘not at all to very much’, 0–10 range) [38]. Satisfaction will be measured at day 1, 4 and 7 post discharge

General evaluations regarding provided care after discharge: patients’ evaluations of their experienced care during the 7th day discharge period are measured using two items: (i) How likely is it that the patient would recommend this hospital to other day care surgery patients (using an adapted item from the CQ index (0–10 ‘would definitely not recommend’ to ‘would definitely recommend’) [39, 40] and (II) their overall rating of the quality of care provided by the hospital during the 7 day discharge period, using an item from the CQ index (0-10 scale, ‘very poor care’ to extremely good care’) [39, 40]. Both items will be measured at discharge days 1, 4 and 7

The quality, experiences and communication regarding remote monitoring with this application will be assed in one by one semi structured interviews. The perceptions of 20 participants are considered satisfactory or when data saturation is reached [41, 42]. See, for interview topics, supplemental material: f1_Interview Topics_version_30_12_2021

#### Other study parameters

The following patient baseline characteristics are collected: age, sex, smoking status, body mass index, ASA classification, PONV score, preoperative NRS, PONV prophylaxis, previous surgical or anaesthesia related complications, history of (chronic) pain, history of motion sickness, surgical specialism, surgical procedure and classification (e.g. laparoscopic cholecystectomy, minor), anaesthesiology technique (i.e. general or spinal anaesthesia), surgery duration, intraoperative medication, PONV and NRS for pain at recovery ward, PONV and NRS for pain at day care ward, complications, duration of admission.

#### Participant timeline {13}

**Fig. 1:**
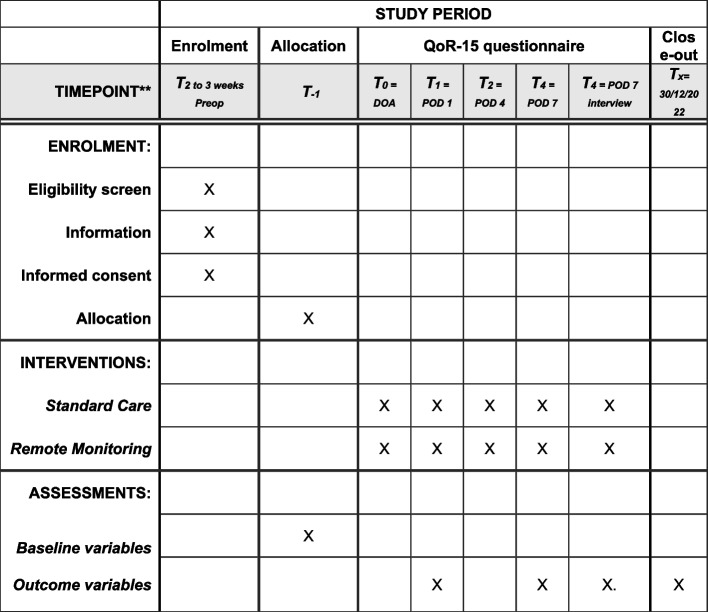
Time schedule of enrolment, intervention and questionnaires. Recommended content SPIRIT 2013 Explanation and Elaboration: T, time point; Preop, preoperative; DOA, day of admission; POD, postoperative day

**Fig. 2:**
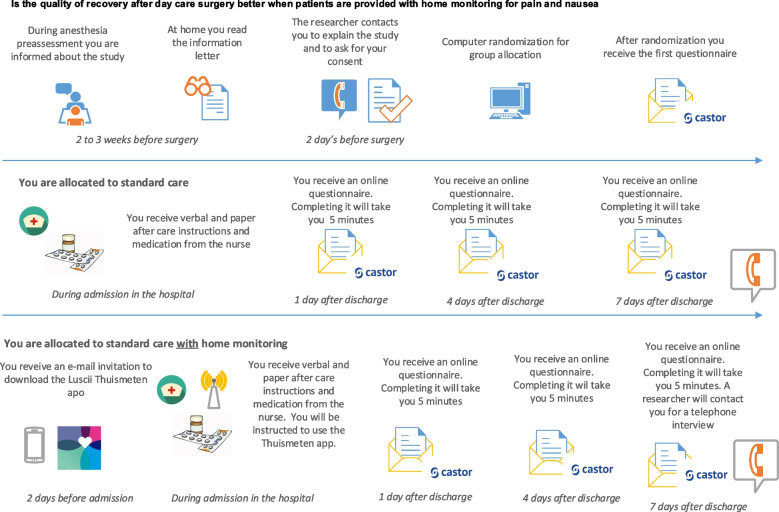
What is the course of the study. From patient information letter

The participant timeline is shown in Figs. 1 and 2

#### Sample size {14}

An 8.0 point difference in the overall score in the QoR-15 questionnaire on postoperative day 7 is considered a minimal clinically relevant difference [34] with standard deviation of 19.1 [43]. Group sample sizes of 91 patients achieve 80% power to detect a difference of − 8.0 with a significance level (alpha) of 0.05 using a two-sided two-sample *t*-test. Sample Size calculation is performed with the PASS 2021 Power Analysis and Sample Size Software. NCSS, LLC. Kaysville, Utah, USA, ncss.com/software/pass. We plan to include 70% extra patients to adjust for dropout or incomplete data and questionnaires. Total sample will be 310 patients divided into two groups.

#### Recruitment {15}

In 2020, under the pressure of the COVID-19 pandemic, over 8000 day care surgical interventions were performed in the participating hospitals. Therefore, we assume that it is likely that the inclusion will be achieved within the intended study period. Eligible patients and reasons for not willing or able to participate in this study are recorded in order to be able to construct a CONSORT flow diagram.

### Assignment of interventions: allocation

#### Sequence generation {16a}

Patients will be allocated to the intervention (remote monitoring) or standard of care group (no remote monitoring) one day before admission for surgery. If a patient is unexpectedly admitted after surgery (and randomization), they are excluded and replaced.

To adjust for practice variation, treatment assignments will be performed in permuted blocks of four, stratified per participating hospital (OLVG Hospital, Maasstad Hospital and Canisius Wilhelmina Hospital). The allocation will be computer-generated with Castor© web application; Castor Services are operated by Ciwit B.V. and located in the Netherlands [44].

#### Concealment mechanism {16b}

Concealment is not applicable.

#### Implementation {16c}

For OLVG hospital researchers, BT, MvE and ER will generate allocation sequence and assign patients to the intervention. For Maasstad hospital, principal investigator JK will be responsible to generate allocation sequence and assign patients to the intervention and for Canisius Wilhelmina; this will be done by hospital nurse specialists LvL and PM.

### Assignment of interventions: blinding

#### Who will be blinded {17a}

Not applicable. This study is not blinded.

#### Procedure for unblinding if needed {17b}

Not applicable. This study is not blinded.

### Data collection and management

#### Plans for assessment and collection of outcomes {18a}

After receiving informed consent, the researchers will record the baseline characteristics to the Castor EDC database and perform the randomization procedure. Intra- and postoperative characteristics and variables will be recorded in Castor EDC by the researchers. Moreover, all data will be de-identified. After randomization, a study identification number will be allocated to all the included patients. The researchers from the participating hospitals will keep a secured database in which patient hospital number corresponds with the study identification number, stored locally at their own hospital server.

#### Plans to promote participant retention and complete follow-up {18b}

Not applicable. It is unlikely according to the design of the study and the intervention that patients will withdraw. In our sample size calculation, we have accounted for incomplete follow-up.

#### Data management {19}

For data collection and validation, an eCRF, including validation checks and appropriate user access rights, will be set up in CASTOR© (www.castoredc.nl). CASTOR© is good clinical practice (GCP) compliant and meets the standard for information security management. The database will be locked as soon as all data are entered, and all discrepant or missing data are resolved—or if all efforts are employed and we consider that the remaining issues cannot be fixed.

#### Confidentiality {27}

All participating hospitals have signed a data processing agreement with Luscii healthtech B.C. that complies with the general data protection regulations (GDPR) and national regulations that apply in the Netherlands. Moreover, all of the patients directly identifying personal data (e.g. name, address, etc.) will be separated from the research data (e.g. measurement data, etc.) and replaced by an assigned code.

#### Plans for collection, laboratory evaluation and storage of biological specimens for genetic or molecular analysis in this trial/future use {33}

Not applicable

### Statistical methods

#### Statistical methods for primary and secondary outcomes {20a}

All statistical analyses will be conducted according to the intention–to–treat principle [45, 46] considering all patients in the treatment groups to which they were randomly assigned, excluding cases lost to follow-up due to withdrawal of consent or cancellation of surgery. Continuous distribution of the data will be assessed by visual inspection of histograms and normality tests. For both study arms, the baseline characteristics will be reported for the intervention and control group and expressed as counts and percentages, means and standard deviations (SD) or medians and interquartile ranges (IQR) whenever appropriate. Baseline characteristics of patients with and without complete follow-up will be compared to examine whether selective dropout occurred. A *p*-value of 0.05 will be considered statistically significant. Analyses will be performed using IBM SPSS Statistics for Windows, Version 22.0. Armonk, NY: IBM Corp.

#### Primary study parameter(s)

The primary outcome, the quality of recovery measured with the QoR-15 at day 7 after day care surgery, will be compared amongst patients randomized to either the intervention and control group, using Student’s *t*-test or a nonparametric Mann-Whitney *U* test.

Mixed effects linear regression models will be conducted to investigate the effect of the smartphone app on QoR-15 over the first week post discharge as a function of the randomization assignment (i.e. intervention vs control), time (i.e. measurements before surgery, and at day 1, 4 and 7 post discharge) and their interaction term. Random effects, i.e. random intercept, will be fitted per individual to account for clustering effects. To adjust for practice variation between the participating hospitals, the factor hospital will be added as fixed effect to the mixed model. To adjust for variation due to socioeconomic status of patients, the factor ‘residential area’ will be added as fixed effect to the mixed model.

#### Secondary study parameter(s)

For the secondary outcomes postoperative pain and PDNV, the same analytical approach will be conducted as proposed for the primary study outcome.

Medication dosage and usage, measured in morphine equivalents; the number of contacts with the hospital, general practitioner or emergency department; the number of surgical complications assessed according to the Clavien-Dindo classification of surgical complications; the number of readmissions emergency department; satisfaction and general evaluation regarding after care will be analysed with descriptive statistics.

Inductive coding will be used to analyse the qualitative data of the interviews evaluating the experienced quality of communication [47]. The audio recordings of the interviews will be transcribed verbatim in Dutch. After familiarization with the data, the transcripts are coded by two researchers (BT and ER). After coding, overarching themes and patterns are identified and labelled within each concept. Analysis are discussed with the research team at several moments.

#### Interim analyses {21b}

Not applicable

#### Methods for additional analyses (e.g. subgroup analyses) {20b}

Subgroup analysis with age- and socio-economic-status groups will be performed to analyse adherence to the e-mail questionnaires and using the smartphone application.

#### Methods in analysis to handle protocol non-adherence and any statistical methods to handle missing data {20c}

Missing data will be diagnosed for its possible systematic relationship between the propensity of data to be missing and values of the data, both missing and observed. If data is missing completely at random (MCAR) or missing at random (MAR), we will apply multiple imputation techniques for the final analysis of the results.

#### Plans to give access to the full protocol, participant level-data and statistical code {31c}

Access to the datasets and statistical code might be available upon an adequately justified request to the corresponding author while maintaining participants’ anonymity.

### Oversight and monitoring

#### Composition of the coordinating Centre and trial steering committee {5d}

All medical specialists, nurses and other hospital personnel involved in perioperative care for surgical day care patients receive information an instruction about the study procedures. The conduct of the study will be performed according to BROK/GCP standards and follow guidelines on improving and standardizing evaluations reports of web-based and mobile health interventions [48].

#### Composition of the data monitoring committee, its role and reporting structure {21a}

The monitoring committee is part of the local research advisory board of OLVG. Monitoring visits will are scheduled for OLVG at initiation, during and at the end of the study. The monitoring committee will report back to the board of directors of OLVG. For Maasstad and Canisius Wilhelmina hospital, researcher BT is responsible for the monitoring visits and will report to local research advisory board of OLVG.

#### Adverse event reporting and harms {22}

As serious adverse events of mobile application usage have not been described so far, we do not expect the need for adverse event reporting However, adverse events will be reported to the medical ethics committee MEC-u 1 month after the end follow-up period of the last included patient. Moreover, adverse events are thoroughly documented and presented in the study manuscript.

#### Frequency and plans for auditing trial conduct {23}

A clinical data management plan will be used to provide high-quality data by adopting standardized procedures to minimize the number of errors and missing data and, consequently, to generate an accurate database for analysis. Accuracy and consistency checks will be carried out by way of validation, pre-specified and ad hoc checking by the researchers. A qualified monitor will be installed to perform study monitoring according to the monitoring plan. Monitoring will be performed to signal early aberrant patterns, issues with consistency, credibility and other anomalies.

#### Plans for communicating important protocol amendments to relevant parties (e.g. trial participants, ethical committees) {25}

All amendments will be reported and need to be approved by the accredited medical ethics committee (METC). We will notify the accredited METC at the end of the study, which is defined as the last patient’s last follow-up.

#### Dissemination plans {31a}

The study protocol is registered before inclusion of the first patient on www.clinicaltrials.gov. The results of the study will find their way into (inter–) national scientific journals and guidelines. We will submit final study findings to peer-reviewed scientific journals in the field of anaesthesiology. A layman’s summary of the results will be published as well and send to the participants if they are interested.

## Discussion

In this study protocol, we describe the design and methods of a multicentre non-blinded randomized trial in which we aim to measure the effects of remote monitoring with a smartphone application on patient recovery after day care surgery. We hypothesize that remote monitoring will contribute to the quality of recovery after day care surgery. Manipulating the aftercare of patients poses methodological, ethical and logistical challenges. To date, standard care in most hospitals after day care surgery is discharging patients with verbal and paper instructions on how to act in case of severe pain, nausea or being worried about their recovery. Being in control with an app and being able to contact the hospital easily should provide patients with a safe feeling and a quicker response on alerts and questions. The success of the study mainly depends on the hospitals responses on patients’ in-app recordings and questions. To ensure communication is standardized, we developed a decision tree and call script to handle alerts and questions. Furthermore, we trained all the medical assistants and researchers involved in how to respond in a similar empathic way.

### Limitations

This trial has several limitations even though it has a randomized design. Due to the design of this study, we have to account for practice variations between the participating centres. We did, however, checked beforehand for large differences and discrepancies and believe that the standard after care delivered in the participating centres is broadly similar to each other. To adjust for differences, we will perform a stratified (per centre) randomization, and mixed effects linear regression models will be conducted to investigate the effect of the smartphone app on QoR-15 over the first week post discharge.

Another limitation is selection bias. Only adult patients with the possession of a smartphone or tablet and able to speak and read Dutch language are able to participate. Moreover, it is likely that only patients being enthusiastic and feeling comfortable in using smartphone applications will join for participation. However, it is likely that we will miss the older patients and ethnic minority groups. Research showed that patients from these groups feel less content to use their mobile phone and eHealth tools [26]. Baseline characteristics of the participating and non-participating patients will be compared to study possible selection bias.

#### Implications for clinical practice

The results from a previous conducted systematic review with the objective to evaluate the effect of perioperative e-Health interventions on the postoperative course, studying 27 unique studies reporting on patient-related outcome [11]. It showed that e-Health leads to similar or improved clinical patient-related outcomes compared to face-to-face perioperative care. However, the quality of included studies was low to moderate. We hope to contribute to the evidence in the field of perioperative e-Health. Facilitating patients and healthcare professionals with a tool for accessible and empathetic communication might lead to an improved quality of the postoperative recovery period, resulting in patients in a faster recovery and being able to return to normal daily activities earlier after the surgical intervention.

## Trial status

This manuscript is based on the QuReMo trial study protocol version 5, 22 June 2022. The Medical Ethics Committees United (MEC-U) approved the study on December 23, 2022. Local approval for the study is obtained in all participating centres before recruitment starts. First inclusion was on April 8, 2022; we planned the last inclusion on September 15, 2022. Trial status at the time of submission is recruiting.

## Supplementary Information

**Additional file 1.** Supplemental material: PIF_IC_e1_e2_QuReMo_V4_22_6_2022.

**Additional file 2.** Supplemental material: k6_discharge information_Dutch.

**Additional file 3.** Supplemental material: Decision tree remote monitoring.

**Additional file 4.** Supplemental material: f1_questionnaire_V1-16-7-2012.

**Additional file 5.** Supplemental material: f1_Interview Topics_version_30_12_2021.

## Data Availability

Study results will be presented at (inter)national meetings and published in a peer-reviewed journal. The datasets used and/or analysed during the conduct of the study will be available from the corresponding author and published as supplemental material along with the primary manuscript. The study protocol was registered before inclusion of the first patient on www.clinicaltrials.gov. A lay summary of the results will be published as well and send to the participants if they are interested.

## Abbreviations

ABR: General Assessment and Registration form (ABR form), the application form that is required for submission to the accredited Ethics Committee; in Dutch: Algemeen Beoordelings- end Registratieformulier (ABR-formulier)
ACWO: Advies Commissie Wetenschap en Onderzoek
AE: Adverse event
App: (smartphone) Application
AR: Adverse reaction
ASA: American Society of Anaesthesiologists
CCMO: Central Committee on Research Involving Human Subjects; in Dutch: Centrale Commissie Mensgebonden Onderzoek
CV: Curriculum vitae
DOA: Day of admission
GCP: Good clinical practice
GDPR: General Data Protection Regulation; in Dutch: Algemene Verordening Gegevensbescherming (AVG)
IC: Informed consent
NRS: Numerical rating scale (for pain)
NVA: Nederlandse vereniging voor Anesthesiologie
MEC-u: Medical Ethics Committees United
METC: Medical research ethics committee (MREC); in Dutch: medisch-ethische toetsingscommissie (METC)
PDNV: Post discharge nausea and vomiting
PDMS: Patient data and management system
POD: Postoperative day
POP: Postoperative pain
Preop: Preoperative
(S)AE: (Serious) adverse event
SPC: Summary of Product Characteristics; in Dutch: officiële productinformatie IB1-tekst
Sponsor: The sponsor is the party that commissions the organization or performance of the research, for example a pharmaceutical company, academic hospital, scientific organization or investigator. A party that provides funding for a study but does not commission it is not regarded as the sponsor but referred to as a subsidizing party
SUSAR: Suspected unexpected serious adverse reaction
UAVG: Dutch Act on Implementation of the General Data Protection Regulation; in Dutch: Uitvoeringswet AVG
WMO: Medical Research Involving Human Subjects Act; in Dutch: Wet Medisch-wetenschappelijk Onderzoek met Mensen
