# Supplementary material for: Quality of recovery after day care surgery with app-controlled remote monitoring: study protocol for a randomized controlled trial
Source: Trials. 2023 Feb 9;24:102. doi: 10.1186/s13063-023-07121-6 (PMC9909143; doi:10.1186/s13063-023-07121-6)
Supplement: Supplementary file 1 — Additional file 1. Supplemental material: PIF_IC_e1_e2_QuReMo_V4_22_6_2022. [file 13063_2023_7121_MOESM1_ESM.pdf]

Translation: Call script home monitoring postoperative pain and nausea after day care surgery

<u>Position</u>: anaesthetist (ANE), physician assistant PA), nurse specialist (NS), medical assistant (MA)

<u>Patients:</u> aged >18 years, after day care surgery

<u>Location:</u> Outpatient department anaesthesia OLVG hospital, - Maasstad hospital, - Canisius Wilhelmina hospital

<u>How:</u> Daily monitoring of patient recordings and alerts in Luscii Dashboard (MA). Patient alerts are algorithm generated (e.g. severe pain, severe nausea, contact with hospital or a question). MA assesses what action and by whom is to be taken.

<u>Actions:</u>

1) No action required e.g. patient recorded a positive positive remark (MA)
2) Contact by text message e.g. answering a question (MA)
3) Contact by telephone (MA)
4) Contact by telephone with anaesthetist / physician assistant or nurse specialist (ANE/PA/NS)

<u>Advice:</u>

-to ease e.g. in case of normal postoperative symptoms but worried (important, re-contact the patient in case of increasing symptoms) (MA/ANE/PA/NS)

-adjust medication (ANE/PA)

-refer to surgical outpatient department or emergency department (MA)

<u>Proceedings and Generic questions telephone contact</u>

1) Open patient file: surgical intervention? Type of anaesthesia? Perioperative course? Number of days after surgery? Earlier contact with hospital? Next appointment?
2) How is the patient at this?
3) Pain, nauseous or other problem?

Pain	Nauseous	Wound	Other problem
• Numerical Rating Scale<br>• Bearable?<br>• Onset?<br>• Where is the pain localized?<br>• When did the pain started?<br>• Increasing?<br>• Charactarisation<br>○ Wound pain<br>○ Burning/ Cold/ Electric shock<br>○ Tingle/ numbness/ Itch<br>○ Recuded sensation to touch<br>○ Increased sensation/pain to touch<br>• 8) Are you hindered?<br>• 9) Other symptoms<br>• 10) Medication what and when?<br>• 11) Is there something you did for relief?<br>• 12) Did you consult somebody for advice?<br>• 13) Are you worried?	• Vomiting?<br>• Onset?<br>• Increasing?<br>• Eating and drinking?<br>• Quantity and colour of urine?<br>• Are you hinderd?<br>• Other symptoms<br>• Medication what and when?<br>• Is there something you did for relief<br>• Did you consult somebody for advice?<br>• Are you worried?	• What is the problem/Symptoms?<br>• Onset?<br>• Sudden swelling?<br>• Red?<br>• Warm?<br>• Pain?<br>• Exudate?<br>• Fever<br>• Other symptoms?<br>• Is there something you did for relief?<br>• Did you consult somebody for advice?<br>• Are you worried?	• What is the problem/Symptoms?<br>• Onset?<br>• Increasing?<br>• Is there something you did for relief<br>• Did you consult somebody for advice?<br>• Are you worried?
Red flags pain	Red flags nausea	Red flags wound	Red flags other problem
• Severe, unbearable pain not responding to medication<br>• Postspinal headache	• Persistent vomiting and unable to eat or drink	• Sudden swelling with painful pressure<br>• Swelling, red and warm, fever	• Chest pain (AP)<br>• Severe dyspnoe<br>• Parese/paralyse
