# Supplementary material for: Quality of recovery after day care surgery with app-controlled remote monitoring: study protocol for a randomized controlled trial
Source: Trials. 2023 Feb 9;24:102. doi: 10.1186/s13063-023-07121-6 (PMC9909143; doi:10.1186/s13063-023-07121-6)
Supplement: Supplementary file 2 — Additional file 2. Supplemental material: k6_discharge information_Dutch. [file 13063_2023_7121_MOESM2_ESM.pdf]

Translation: Aftercare instructions day care ward

You underwent a surgical procedure during a day care admission in our hospital. In this letter, you will find instructions for your recovery at home.

After surgery

After most interventions, you are allowed to eat and drink. If indicated differently, your doctor or nurse will provide you with tailored instructions.

It is important to be active after the intervention; regular resting periods during your recovery are not advised. If indicated differently, your doctor or nurse will provide you with tailored instructions

Wound care

- Showering: The first day after the intervention, you are allowed to take a shower

□ Remove the pressure bandage before you take a shower

□ Do not remove adhesive strips or transparent wound dressing. They will release themselves a few days after the intervention. You are allowed to take a shower

□ Remove your wound dressing during the shower

- After the shower carefully pat dry the wound, and leave it two minutes to dry
- Only use a new wound dressing if there is still exudate

Sutures

□ Sutures will dissolve

□ Sutures will be removed after two week on the outpatient department

- Taking a bath, swimming or visiting the sauna is allowed two weeks after the intervention and when the wound is healed.

Pain management

Pain management is important for your recovery. On the day of the intervention and the first day after, we advise you to take your analgesics according to the schedule below. If the second day after the intervention your pain is decreasing you can try to taper off your analgesics.

Medication

<table>
<tr><td>Analgesics</td><td>Date</td><td>Time</td></tr>
<tr><td>Acetaminophen 1000mg</td><td></td><td><table><tr><td>6:00</td><td>12:00</td><td>18:00</td><td>24:00</td></tr></table></td></tr>
<tr><td>…</td><td></td><td></td></tr>
<tr><td>…</td><td></td><td></td></tr>
<tr><td>…</td><td></td><td></td></tr>
</table>

Complications

After all surgical interventions there is a small chance medical complications occurs. You are advised to contact the hospital when you experience one of the following symptoms

Fever above 38,5 degrees Celsius

Unbearable and increasing pain

Persisting blood loss from the wound

Wound infection: red, warm, swelling and exudate (pus)

If the wound shows no signs of healing after two weeks

Follow-up appointment

□ You receive an appointment for your follow-up from the nurse

□ We will send you a letter for your follow-up appointment

□ You have to make a follow-up appointment yourself

□ A follow up appointment is not necessary

Exceptions and particularities

……………………………………………….

……………………………………………….

……………………………………………….

……………………………………………….

……………………………………………….
