# Supplementary figures and images for: Quality of recovery after day care surgery with app-controlled remote monitoring: study protocol for a randomized controlled trial
Source: Trials. 2023 Feb 9;24:102. doi: 10.1186/s13063-023-07121-6 (PMC9909143; doi:10.1186/s13063-023-07121-6)

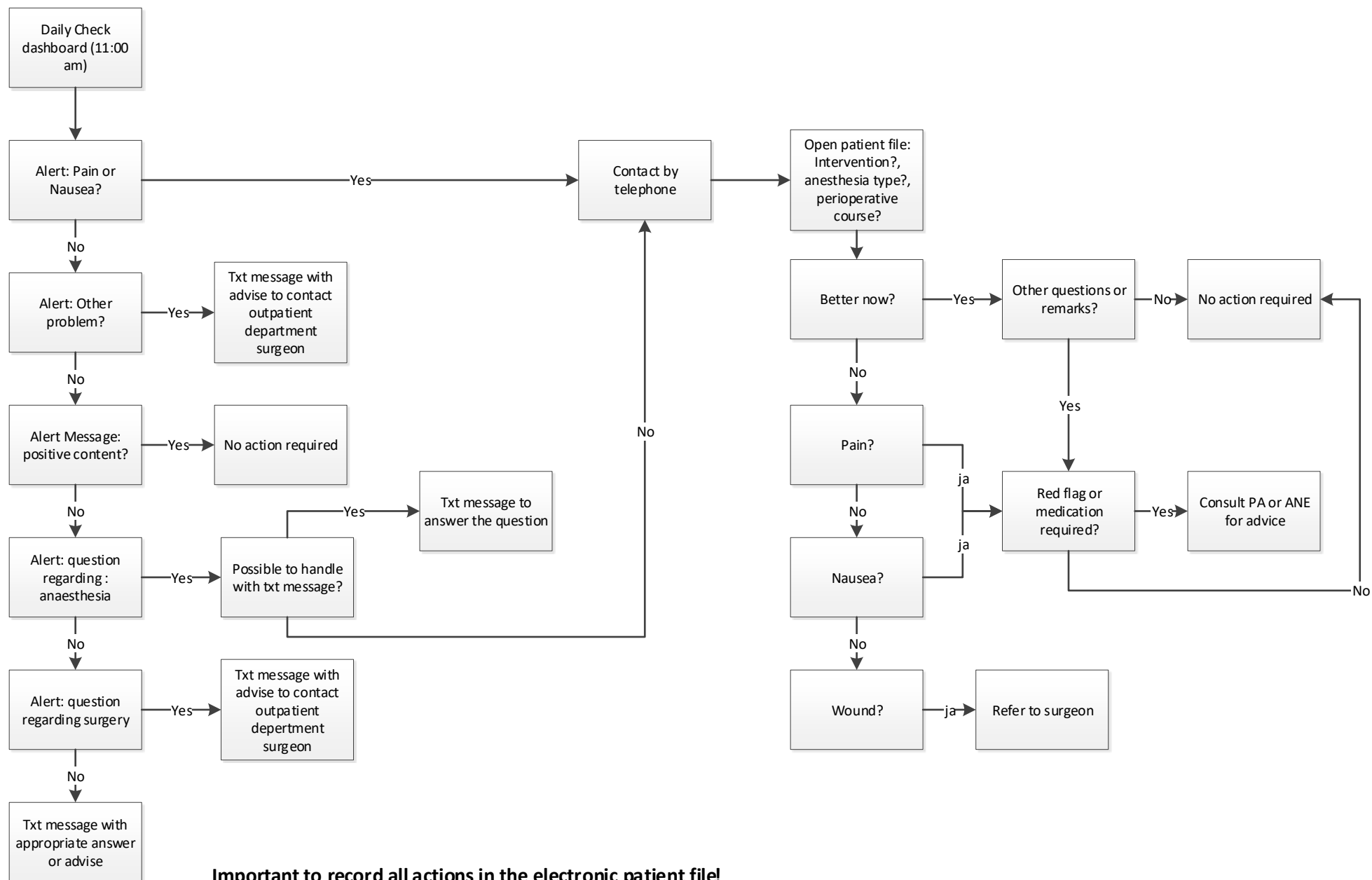

Supplement: Supplementary file 3 — Additional file 3. Supplemental material: Decision tree remote monitoring. [file 13063_2023_7121_MOESM3_ESM.pdf]
