# Supplementary material for: Quality of recovery after day care surgery with app-controlled remote monitoring: study protocol for a randomized controlled trial
Source: Trials. 2023 Feb 9;24:102. doi: 10.1186/s13063-023-07121-6 (PMC9909143; doi:10.1186/s13063-023-07121-6)
Supplement: Supplementary file 4 — Additional file 4. Supplemental material: f1_questionnaire_V1-16-7-2012. [file 13063_2023_7121_MOESM4_ESM.pdf]

**Questionnaire: Quality of Recovery after day care surgery with app controlled Remote Monitoring**

Study no.

Hospital no.

Randomisation no.

Date: - - / - - / - -

**Before admission:** ☐

**After discharge:** ☐

**PART A**

**How have you been feeling in the last 24 hours?**

**(0 to 10, where: 0 = none of the time [poor] and 10 all of the time [excellent]**

1. Able to breathe easily.

| | | | | | | | | | | | | |
|---|---|---|---|---|---|---|---|---|---|---|---|---|
| Non of the time [poor] | 0 | 1 | 2 | 3 | 4 | 5 | 6 | 7 | 8 | 9 | 10 | All of the time [excellent] |

2. Been able to enjoy food.

| | | | | | | | | | | | | |
|---|---|---|---|---|---|---|---|---|---|---|---|---|
| Non of the time [poor] | 0 | 1 | 2 | 3 | 4 | 5 | 6 | 7 | 8 | 9 | 10 | All of the time [excellent] |

3. Feeling rested.

| | | | | | | | | | | | | |
|---|---|---|---|---|---|---|---|---|---|---|---|---|
| Non of the time [poor] | 0 | 1 | 2 | 3 | 4 | 5 | 6 | 7 | 8 | 9 | 10 | All of the time [excellent] |

4. Have had a good sleep.

| | | | | | | | | | | | | |
|---|---|---|---|---|---|---|---|---|---|---|---|---|
| Non of the time [poor] | 0 | 1 | 2 | 3 | 4 | 5 | 6 | 7 | 8 | 9 | 10 | All of the time [excellent] |

5. Able to look after personal toilet and hygiene unaided.

| | | | | | | | | | | | | |
|---|---|---|---|---|---|---|---|---|---|---|---|---|
| Non of the time [poor] | 0 | 1 | 2 | 3 | 4 | 5 | 6 | 7 | 8 | 9 | 10 | All of the time [excellent] |

6. Able to communicate with family or friends.

| | | | | | | | | | | | | |
|---|---|---|---|---|---|---|---|---|---|---|---|---|
| Non of the time [poor] | 0 | 1 | 2 | 3 | 4 | 5 | 6 | 7 | 8 | 9 | 10 | All of the time [excellent] |

7. Getting support from hospital doctors and nurses.

| | | | | | | | | | | | | |
|---|---|---|---|---|---|---|---|---|---|---|---|---|
| Non of the time [poor] | 0 | 1 | 2 | 3 | 4 | 5 | 6 | 7 | 8 | 9 | 10 | All of the time [excellent] |

8. Able to return to work or usual home activities.

| | | | | | | | | | | | | |
|---|---|---|---|---|---|---|---|---|---|---|---|---|
| Non of the time [poor] | 0 | 1 | 2 | 3 | 4 | 5 | 6 | 7 | 8 | 9 | 10 | All of the time [excellent] |

9. Feeling comfortable and in control.

| | | | | | | | | | | | | |
|---|---|---|---|---|---|---|---|---|---|---|---|---|
| Non of the time [poor] | 0 | 1 | 2 | 3 | 4 | 5 | 6 | 7 | 8 | 9 | 10 | All of the time [excellent] |

10. Having a feeling of general well-being.

| | | | | | | | | | | | | |
|---|---|---|---|---|---|---|---|---|---|---|---|---|
| Non of the time [poor] | 0 | 1 | 2 | 3 | 4 | 5 | 6 | 7 | 8 | 9 | 10 | All of the time [excellent] |

**PART B**

**Have you had any of the following in the last 24 hours?**

**(0 to 10, where: 10 = none of the time (excellent) and 0 = all of the time [poor])**

1. Moderate pain.

| | | | | | | | | | | | | |
|---|---|---|---|---|---|---|---|---|---|---|---|---|
| Non of the time [excellent] | 10 | 9 | 8 | 7 | 6 | 5 | 4 | 3 | 2 | 1 | 0 | All of the time [poor] |

2. Severe pain.

| | | | | | | | | | | | | |
|---|---|---|---|---|---|---|---|---|---|---|---|---|
| Non of the time [excellent] | 10 | 9 | 8 | 7 | 6 | 5 | 4 | 3 | 2 | 1 | 0 | All of the time [poor] |

3. Nausea or vomiting.

| | | | | | | | | | | | | |
|---|---|---|---|---|---|---|---|---|---|---|---|---|
| Non of the time [excellent] | 10 | 9 | 8 | 7 | 6 | 5 | 4 | 3 | 2 | 1 | 0 | All of the time [poor] |

4. Feeling worried or anxious.

| | | | | | | | | | | | | |
|---|---|---|---|---|---|---|---|---|---|---|---|---|
| Non of the time [excellent] | 10 | 9 | 8 | 7 | 6 | 5 | 4 | 3 | 2 | 1 | 0 | All of the time [poor] |

5. Feeling sad or depressed.

| | | | | | | | | | | | | |
|---|---|---|---|---|---|---|---|---|---|---|---|---|
| Non of the time [excellent] | 10 | 9 | 8 | 7 | 6 | 5 | 4 | 3 | 2 | 1 | 0 | All of the time [poor] |

**PART C**

**Please answer or indicate the following questions**

1. Please indicate the number of contacts with the hospital, your general practioner or other healthcare institutes, regarding your recovery the past 24 hours.

2. Please indicate if a problem occured the past 24 hours regarding the recovery.
   If Yes, what was the problem:

3. Please indicate if a re-admission to hospital or emergency department was necessary.
   If yes, indicate which institution:

4. Please indicate your satisfaction with the provided care during your recovery

| | | | | | | | | | | | | |
|---|---|---|---|---|---|---|---|---|---|---|---|---|
| Not at all [poor] | 0 | 1 | 2 | 3 | 4 | 5 | 6 | 7 | 8 | 9 | 10 | Very much [excellent] |

5. Please indicate your trust in the provided care during your recovery

| | | | | | | | | | | | | |
|---|---|---|---|---|---|---|---|---|---|---|---|---|
| Not at all [poor] | 0 | 1 | 2 | 3 | 4 | 5 | 6 | 7 | 8 | 9 | 10 | Very much [excellent] |

6. How likely is it that you would recommend this hospital to other patients in need of day care surgery?

| | | | | | | | | | | | | |
|---|---|---|---|---|---|---|---|---|---|---|---|---|
| Not at all [poor] | 0 | 1 | 2 | 3 | 4 | 5 | 6 | 7 | 8 | 9 | 10 | Very much [excellent] |

7. Any other remarks or suggestions:

Thank you for your assistance

Please check that all questions have been answered.
