# Supplementary material for: Quality of recovery after day care surgery with app-controlled remote monitoring: study protocol for a randomized controlled trial
Source: Trials. 2023 Feb 9;24:102. doi: 10.1186/s13063-023-07121-6 (PMC9909143; doi:10.1186/s13063-023-07121-6)
Supplement: Supplementary file 5 — Additional file 5. Supplemental material: f1_Interview Topics_version_30_12_2021. [file 13063_2023_7121_MOESM5_ESM.pdf]

Interview Topics QuReMo Trial: NL78144.100.21

Dutch

De kwaliteit van herstel na een dagbehandeling operatie met thuismonitoring

1) Heeft thuismonitoring via een app bijgedragen aan uw lichamelijk herstel?

2) Heeft thuismonitoring via een app bijgedragen aan uw emotioneel herstel?

   a. Voelde u zich gesteund door het gebruik van de app?

3) Heeft het contact met het zorgpersoneel door het gebruik van de app, via berichten of tijdens een telefonisch gesprek bij gedragen aan uw lichamelijk en emotioneel herstel?

   a. Was het zorgpersoneel voldoende meelevend en positief?

4) Hoe voelt het om persoonlijke informatie via de app te delen?

5) Hoe ervaart u het om regelmatig notificaties in de app te ontvangen voor het registreren van pijn en misselijkheid?

6) Hoe kunnen we het contact via de app tussen patiënten en het zorgpersoneel verbeteren?

English

Quality of recovery after day care surgery with app controlled Remote Monitoring

1) Has remote monitoring with an app contributed to your physical recovery?

2) Has remote monitoring with an app contributed to your emotional recovery?
   a. Did you feel supported by using the app?

3) Has the contact with care professionals, through messaging or by telephone contributed to your physical and emotional recovery?
   a. Were the care professionals compassionate and positive enough?

4) How do you feel about sharing personal information in the app?

5) How do you feel about being confronted regularly with notifications in the app to record you pain and nausea?

6) How can we improve the contact via the app between patients and care professionals?
